# Qualitative and Quantitative Mass Spectrometry in Salivary Metabolomics and Proteomics

**DOI:** 10.3390/metabo13020155

**Published:** 2023-01-20

**Authors:** Paulina Grocholska, Marta Kowalska, Remigiusz Bąchor

**Affiliations:** Faculty of Chemistry, University of Wroclaw, F. Joliot-Curie 14, 50-383 Wroclaw, Poland

**Keywords:** saliva, metabolomics, proteomics, LC-MS, quantitative analysis, isobaric labelling reagents, ionisation enhancers, isotopically labelled standards

## Abstract

The metabolomics and proteomics analysis of saliva, an excellent biofluid that is a rich source of biological compounds, allows for the safe and frequent screening of drugs, their metabolites, and molecular biomarkers of various diseases. One of the most frequently used analytical methods in saliva analysis is liquid chromatography coupled with mass spectrometry (LC-MS) and tandem mass spectrometry. The low ionisation efficiency of some compounds and a complex matrix makes their identification by MS difficult. Furthermore, quantitative analysis by LC-MS frequently cannot be performed without isotopically labelled standards, which usually have to be specially synthesised. This review presented reports on qualitative and quantitative approaches in salivary metabolomics and proteomics. The purpose of this manuscript was to present the challenges, advances, and future prospects of mass spectrometry, both in the analysis of salivary metabolites and proteins. The presented review should appeal to those interested in the recent advances and trends in qualitative and quantitative mass spectrometry in salivary metabolomics and proteomics, which may facilitate a diagnostic accuracy, the evaluation of treatment efficacy, the early diagnosis of disease, and a forensic investigation of some unapproved drugs for any medical or dietary administration.

## 1. Introduction

**Figure 1:**
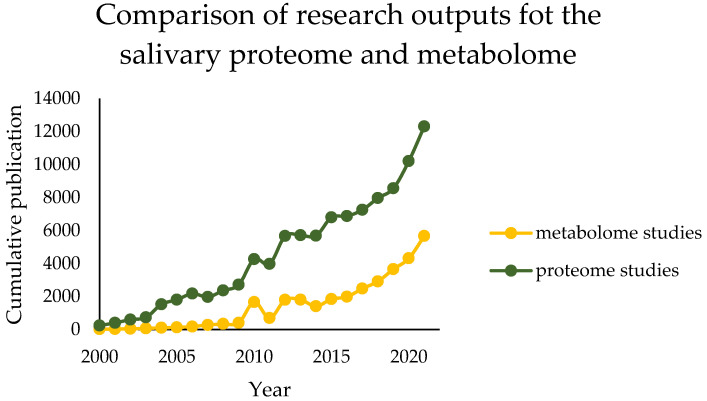
A comparison of research outputs for salivary proteomic and metabolomic studies. Data were gathered by searching Google Scholar for the terms “saliva” OR “salivary” AND “proteome” OR “proteomics” (green line) compared to “saliva” OR “salivary” AND “metabolome” OR “metabolomics” (yellow line) in article title fields.

The assay of saliva specimens is an increasing area of research with implications for both basic and clinical purposes. It provides important information regarding the functioning of various organs within the body [1]. Saliva can also be collected through a simple and non-invasive method without medical supervision. These features make it possible for its application to monitor several biomarkers in humans. As a diagnostic tool, it will also be beneficial to patients suffering from clotting disorders such as haemophilia and in patients with compromised venous access [2,3,4,5]. There are several preliminary studies with promising results that demonstrate that saliva can be used to detect lung cancer, pancreatic cancer, breast cancer, and type II diabetes [6,7,8]. To bring it to a clinical reality, there is a need for a further scientific validation for each disease, and it is also required to benchmark the diagnostic capacity of saliva against other bodily fluids. There are numerous scientific publications appearing in large numbers, especially since 2005. Although salivary metabolomics is not well-developed, it has shown a relatively high growth in the last few years (Figure 1) [9].

### 1.1. Salivary Metabolomics for Drug, Hormones, and Tissue Metabolites Analysis

Similar to other -omic sciences, metabolomics is a technology-driven discipline. Analytical chemistry is constantly evolving and taking advantage of new developments in analytical techniques, instrumentation, analytical software, statistical methods, or computational techniques to accelerate or improve data collection, analysis, and interpretation. The primary analytical technologies used in metabolomics include liquid chromatography coupled with single-stage mass spectrometry (LC-MS) [10,11,12] or tandem mass spectrometry (LC-MS/MS) [13,14,15,16,17,18], gas chromatography coupled to mass spectrometry (GC-MS) [10,11,19,20], high- or ultrahigh-performance liquid chromatography coupled to UV or fluorescent detection (HPLC/UPLC) [21,22,23,24,25,26,27,28], and nuclear magnetic resonance (NMR) spectroscopy [29,30,31,32,33,34,35,36]. Each analytical platform has its advantages and disadvantages. The choice of the platform depends primarily on the focus of the study, as well as on the nature of the samples [37]. However, the selection of a given platform is often also determined by the cost, its accessibility, and the available expertise. It is important to remember that there is no single analytical platform that can perform a complete identification and quantification of all metabolites for a typical biological sample. The best metabolomic studies often employ multiple technology platforms. Today, LC-MS, GC-MS, and NMR spectroscopy are the commonly used analytical methods in metabolomics. While LC-MS and GC-MS methods are becoming increasingly popular (accounting for over 80% of published metabolomics studies to date), there is still considerable interest in using NMR-based methods for metabolomics studies [38]. The relative ease of the sample preparation, the ability to quantify the metabolite levels, the high level of experimental reproducibility, and the inherently non-destructive nature of NMR spectroscopy have made it the preferred platform for long-term or large-scale clinical metabolomic studies. These advantages, however, are often outweighed by the fact that most other analytical techniques, including both LC-MS and GC-MS, are inherently more sensitive than NMR, with the lower limits of detection typically being 10 to 100 times better [38]. Moreover, the complex biofluid spectra often exhibit an extensive signal dynamic range, accompanied by the severe overlap between metabolites and macromolecules. Thus, an unambiguous assignment often becomes tedious or impossible. The precise identification of chemical moieties, the spin-system connectivity, and, finally, the molecules’ chemical identity and abundance are essential for analysing the mixtures of metabolites [38]. The assignment is the critical step for metabolite identification, which requires the recording of multiple datasets. The work is accompanied by high-throughput data from numerous samples of biofluids, followed by several multivariate data analyses [39]. The workflow’s precise aim becomes identifying the biomarkers by monitoring specific changes in the biofluid metabolite composition. The main challenge in the targeted analysis is the presence of large macromolecules such as proteins, lipids, and membranes that cover the signals from the small metabolites [40]. 

Liquid chromatography NMR (i.e., LC-NMR) is not necessarily a new hybrid NMR technique, but the trend towards hybrid NMR techniques in metabolomics is new and important as it greatly enhances the capabilities of NMR-based metabolomics. Combining liquid chromatography with NMR spectroscopy (i.e., LC-NMR) allows one to take advantage of the strengths of modern (HPLC, UPLC) chromatographic separation techniques to greatly simplify the complexity of biological mixtures. The spectral complexity of biofluids such as urine or faecal water extracts is so great that it is not possible to identify or quantify many of the compounds detected via 1D or 2D NMR spectroscopy [41]. Other hybrid NMR techniques also exist, such as LC-SPE-NMR, where LC-NMR is combined with solid phase extraction (SPE). This approach can help address some of the aforementioned limitations of LC-NMR and can significantly improve metabolite or natural product identification [42]. LC-NMR can also be combined with mass spectrometry (LC-NMR-MS) to perform a structure elucidation of novel compounds [43].

#### 1.1.1. Salivary Cortisol Analysis

Recently, salivary cortisol has received much attention as a biomarker of psychological stress, where the levels may be related to mental or physical diseases [44]. Salivary cortisol and cortisone have been considered useful targets in the evaluation of mineralocorticoid or glucocorticoid excess, congenital adrenal hyperplasia (CAH), and adrenal insufficiency. Cortisol is the main glucocorticoid in humans and is increased in Cushing’s syndrome or decreased in Addison’s disease [45,46,47]. Cortisol can also be associated with cardiovascular disease [48] and anti-inflammatory and immunosuppressive effects [49,50]. It has been found that salivary free cortisol is a good indicator of unbound concentrations of cortisol in serum or plasma. [51,52,53,54,55,56]. Thus, a sensitive and specific analytical tool is needed to determine the low range of cortisol and cortisone in saliva. The cortisol concentration in the saliva is about 10 times lower than that of the cortisol in plasma or serum. Various quantitative methods have been developed for the measurement of salivary cortisol by using internal standards, deuterated standards, or calibration curves [9,53,57,58]. Among them, liquid chromatography–tandem mass spectrometry (LC-MS/MS) has been considered to provide a fast, highly sensitive, and specific method for steroid quantification in clinical samples, allowing for the simultaneous analysis of different steroids. LC-MS/MS also provides the specificity needed to overcome an interference by similar steroids. The salivary cortisol and cortisone levels in an English population were recently reported using LC-MS/MS [55].

Lee et al. [59] measured the levels of salivary cortisol and cortisone in Korean adults using liquid chromatography–tandem mass spectrometry (LC-MS/MS) and cortisol-d_4_ as an internal standard. The salivary cortisol and cortisone were separated within 10 min. The regression coefficients (r) of the calibration curves were greater than 0.999 for the two steroids. The limits of quantitation (LOQ) were 0.2 ng/mL for cortisol and 1 ng/mL for cortisone. The intraday precision of the assay was <3.9% and 8.6% for cortisol and cortisone, respectively, and the inter-day precision was <1.9% and 4.3% for cortisol and cortisone, respectively. The salivary cortisone concentrations were approximately 4–9 times higher than those of the salivary cortisol during the daytime. Diurnal rhythms, during which the cortisol and cortisone concentrations were higher in the morning than in the afternoon, were also observed. This assay may be useful for the diagnosis of several adrenal dysfunctions in clinical biochemistry.

#### 1.1.2. Salivary *p*-Cresol Sulphate and Indoxyl Sulphate

Giebułtowicz et al. [60] developed and validated a high-performance liquid chromatography–tandem mass spectrometry (LC-MS/MS) method for the analysis of *p*-cresol sulphate and indoxyl sulphate in saliva using *p*-cresol sulphate-d_7_ and indoxyl sulphate-d_4_. The concentration of these toxins in serum is associated with the stage of renal failure. They observed a tendency for an increase in the concentration of toxins in saliva in the elderly. The condition could be a result of age-related diseases, such as diabetes and kidney function decline. The method was validated for linearity, precision, accuracy, stability (freeze/thaw stability, stability in autosampler, short- and long-term stability, stock solution stability), dilution integrity and matrix effect. The analysed validation criteria were fulfilled. The results encourage validation of saliva as a clinical sample for monitoring toxin levels in organisms.

#### 1.1.3. Analysis of Steroids

A selective and sensitive LC-MS/MS method (using multiple reaction monitoring upon electrospray ionisation) was developed for steroids (androstenedione, cortisol, cortisone, 11-deoxycortisol, 21-deoxycortisol, 17α-hydroxyprogesterone, and testosterone) in saliva for the diagnosis and treatment of disorders like congenital adrenal hyperplasia, Cushing’s syndrome or Addison’s disease [61].

Gao et al. [62] developed a method for measuring estradiol and six other steroid hormones in human saliva using high-performance liquid chromatography–tandem mass spectrometry (LC–MS/MS) with Atmospheric Pressure Chemical Ionisation (APCI) coupled with online solid phase extraction (SPE) and application of deuterated standards of all the analysed compounds.

#### 1.1.4. Analysis of Creatinine

Saliva can be used as a non-invasive alternative to serum for creatinine estimation in monitoring renal function. The normal range of serum creatinine, determined by the Jaffe test [63], is 0.6–1.5 mg/dL [64] and salivary creatinine is 0.05–0.2 mg/dL [65]. Venkatapathy et al. [66] observed a significantly high creatinine level in CKD patients’ serum and saliva compared with controls. A similar observation was made by Davidovich et al. [67]. This is because the kidneys are unable to excrete creatinine in renal failure, and hence its concentration in blood increases [64]. This may be due to an increased serum creatinine concentration, which creates an increased concentration gradient, which in turn increases the diffusion of creatinine from serum to saliva in CKD patients [68]. The determination of creatinine concentration in biological samples may also be performed by applying isotope-labelled standards of this metabolite, including commercially available Cre-d_3_ and Cre-d_5_ which are expensive due to the complicated synthesis. The price of the used internal standard and the total cost of the analysis are the main factors limiting the possibilities of many research groups in their use. Additionally, due to the possible isotopic effect of deuterium, associated with the lack of isotopologues co-elution, their application in quantitative metabolomics can be limited. Therefore, there is a strong need to develop a new, simple and inexpensive methods of isotopically labelled internal standard preparation. 

Recently, Bąchor et al. [69] used the liquid chromatography–mass spectrometry (LC-MS) method with an isotopically labelled analogue of creatinine as an internal standard for quantifying the creatinine concentration in urine. This method of preparation of deuterated analogues of creatinine, via the H/D exchange of hydrogens located at the α-carbon (α-C) of the *N*-methylated amino acid part, under basic conditions may be successfully used for the creatinine concentration in saliva.

#### 1.1.5. Antiepileptic Drugs

Other interesting compounds found in saliva are antiepileptic drugs, which may have clinical application. Greenaway et al. [70] examined the relationship between serum and saliva lacosamide concentrations to determine whether saliva may be a useful alternative to serum for therapeutic drug monitoring. Mecarelli et al. [71] investigated the possible correlation between saliva and serum levetiracetam concentrations based on the calibration curve. These results indicate that levetiracetam, like other AEDs and nootropic drugs, can be measured in saliva as an alternative to blood-based assays. The results of the group of Mierzejewski [72,73] tend to indicate that saliva may be a reliable alternative to plasma for monitoring the levetiracetam concentrations too. Similar results were developed and validated for the determination of levetiracetam in saliva by using a simple isocratic HPLC method based on simple protein precipitation by Hamdan et al. [74]. Since racetams, a wide category of synthetic nootropics, are popular supplements for boosting cognition, which may cause side effects, there is a strong need for the development of methods of their quantification. 

**Figure 2:**
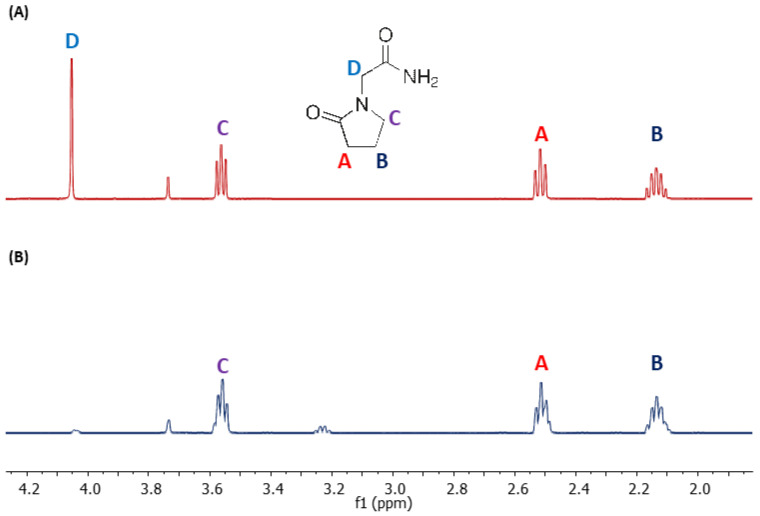
^1^H NMR spectrum of (**A**) piracetam in D_2_O and (**B**) piracetam after 72 h incubation in the mixture of 1% N,N,N-triethylamine solution in D_2_O, lyophilisation and redissolving in D_2_O. The letter designations of the carbons correspond to the signals marked with these letters in the NMR spectrum.

Recently, we developed a method for the preparation of α-carbon deuterated piracetam, oxiracetam, nefiracetam, and phenylpiracetam via HDX in the presence of D_2_O and *N,N,N*-triethylamine as a catalyst. The exchange reaction kinetics were analysed by mass spectrometry and NMR techniques (Figure 2). The obtained results revealed that the reaction rate depends on the chemical structure of the used racetam with the highest extent for oxiracetam and piracetam. The NMR analysis clearly indicated the places of the ongoing exchange process. An LC-MS analysis of a mixture of deuterated and non-deuterated isotopologues of analysed racetams revealed their co-elution. The obtained data clearly presents the applicability of the proposed deuterated standards of model racetams in their quantitative analysis by LC-MS. 

**Figure 3:**
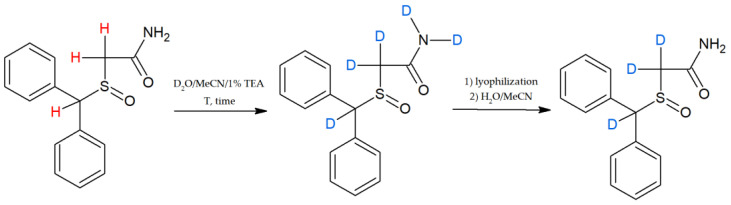
Schematic reaction of hydrogen–deuterium exchange for armodafinil [75].

Another nootropic drug, which can be detected from saliva, is armodafinil. Grocholska et al. [75,76] demonstrated the possibility of the preparation of a deuterated armodafinil standard via a base-catalysed hydrogen–deuterium exchange (HDX) at the carbon atom and the applicability of the obtained standard in the quantitative analysis of armodafinil in human urine samples by LC-MS (Figure 3). This method can be successfully used for saliva samples. We also reported our investigation on the preparation of deuterated standards of denatonium benzoate [77] and cyclosporine A [78] based on the hydrogen–deuterium exchange of carbon-bounded hydrogen atoms in the presence of *N,N,N*-triethylamine as a catalyst.

##### Carbamazepine

Dziurkowska et al. [79] developed a method of saliva purification that would enable the determination of carbamazepine and its metabolite, carbamazepine-10,11 epoxide. They found that the method developed is rapid, sensitive, reliable, and can be used to monitor the concentration of carbamazepine and metabolites in patients’ saliva.

#### 1.1.6. Soluble Tumour Necrosis Factor-Related Apoptosis-Inducing Ligand (sTRAIL)

The soluble tumour necrosis factor-related apoptosis-inducing ligand (sTRAIL) induces apoptosis via the extrinsic death receptor pathway and may be a biomarker in the pathogenesis of a broad range of diseases. To investigate the role of sTRAIL in asthma, Wilffert et al. [80] developed a quantitative LC–MS/MS method with a lower limit of quantitation (LLOQ) of ≈3 pM in induced sputum (174 pg/mL) and saliva (198 pg/mL) without the use of antibodies. The method was validated regarding the stability, accuracy, and precision using the standard addition approach and fully metabolically ^15^N-labelled hrTRAIL as the internal standard. The results indicate that it is possible to quantify cytokines such as sTRAIL at the pM level by LC–MS/MS without the use of antibodies.

### 1.2. Metabolomics Profiling of the Salivary Microbiome

The salivary metabolome has been examined in a wide range of conditions of sampling and disease status [81]. However, there are fewer which have specifically addressed the contribution of the microbiome to the salivary metabolome. While it may be difficult to deconvolute the host response and the microbial contributions to the salivary metabolome, there have been some studies that have reported changes which may contribute to our understanding of the oral microbiome-derived metabolome to saliva. 

Marchesan et al. [82] explored the change in the salivary metabolome before and after 21 days of experimental biofilm overgrowth in donors in five categories of periodontal status. The authors identified 272 microbial species in the plaque samples from the biofilm overgrowth sites. The authors observed that the microbes were highly associated with cyclodipeptides cyclo (-leucine-proline) and cyclo (-phenylalanine-proline), and these microbes and metabolites were correlated with microbial communities associated with more severely affected periodontal disease biofilm donors. The finding of these cyclodipeptides was unexpected by the authors, and they speculated as to the functionality of these molecules in quorum-sensing, the control of virulence factors, and the inhibition of the growth of commensal organisms to the benefit of the emergence of pathobionts.

Bregy et al. [83] compared the volatile metabolites found in the headspace above bacterial cultures of *Ag. Actinomycetemcomitans, Po. Gingivalis, Ta. Forsythia*, and *Tr. Denticola* with those that could be found in the saliva from either a patient with severe periodontitis or two healthy control donors. The authors found 120 metabolites in the bacterial cultures. *Po. gingivalis, Ta. forsythia,* and *Tr. denticola* were detected in the patient with periodontitis but not in the control donors. The mass spectrometry technique used, secondary electrospray ionisation, is often used to derive the volatile metabolic fingerprints of individual species and so may be able to pinpoint the contributions of bacteria to the complex mixture of saliva. An in-depth analysis of a much wider range of oral microbes would help in a further elucidation.

### 1.3. Conclusions and Perspectives

Recently, salivary metabolomics has become an extremely dynamically developing science that can help solve many problems in the field of clinical diagnosis or forensic sciences by developing methods of an ultrasensitive identification and quantitative analysis of molecular biomarkers (Table 1). 

Despite many successes and innovative solutions, a constant problem in this field is the limited possibility of identifying many components, their quantification, and the elimination of matrix effects, which makes it difficult or impossible to conduct an unambiguous analysis. Many of these challenges are bravely faced by proteomics, whose range of tools and possibilities is slightly wider, due to more extensive research in this field. Until relatively recently, the metabolite composition of saliva has been largely overlooked or treated as a sidenote to protein composition. Despite this, there has been an increase in salivary metabolomic research in recent years [9].

Since the metabolomics and proteomics integration may provide a systematic perspective on the dynamic changes in proteins and metabolites, this review presents also salivary proteomics approaches. 

## 2. Salivary Proteome

The salivary proteome can be defined as the complete set of proteins present in the whole saliva at any given time, regardless of its origin. In saliva, the main sources of proteins are salivary glands; however, blood, oral tissues, and microorganisms (in particular bacteria) could also be important components and should be considered as a part of the salivary proteome [84].

Saliva is an extremely attractive diagnostic target in the search for potential disease biomarkers due to the simple, safe, non-invasive, and relatively inexpensive collection compared to blood sampling [85]. The saliva secreted from salivary glands and the gingival crevice has several plasma-derived components in an almost 20–30% overlap with the plasma proteome. Most of these components have enzymatic functions and antimicrobial and transport activity [86]. Moreover, the 22 most abundant proteins in plasma represent 99% of the total protein content, making the identification and quantification of the remaining proteins very challenging [85]. 

The analysis of changes in the proteins concentrations in response to different pathophysiological states can provide a better understanding of complex biological processes and can lead to the discovery of new potential biomarkers of disease states [87]. The salivary proteome has been described in several diseases, including oral leukoplakia and oral squamous cell carcinoma [88], chronic graft-versus-host disease Sjögren’s syndrome [89], and other autoimmune disorders such as SAPHO, bipolar disorder, schizophrenia and genetic diseases such as Wilson disease, and Down syndrome [90,91].

### 2.1. Methods of the Salivary Proteome Analysis

The human salivary proteome contains numerous proteins within a wide concentration range, which makes it an attractive diagnostic target. However, it may also contribute to making analysis challenging [92]. A variety of analytical methods have been used to analyse and characterise the salivary proteome, including gel electrophoresis [93], liquid chromatography (LC) or two-dimensional liquid chromatography (2D-LC) [94], capillary electrophoresis (CE) [91], nuclear magnetic resonance spectroscopy [95,96], mass spectrometry (MS) [97], and immunological tests [98]. Three commonly used techniques are two-dimensional gel electrophoresis (2D-E), liquid chromatography–mass spectrometry (LC-MS), matrix-assisted laser desorption ionisation–time of flight/mass spectrometry (MALDI-TOF/MS), and surface-enhanced laser desorption/ionisation–time-of-flight mass spectrometry (SELDI-TOF/MS) [99]. 

Proteome may be characterised with the use of two approaches: top-down and bottom-up proteomics. In a bottom-up approach, proteins are digested into peptides, then an MS measurement is performed where the resulting peptides are detected and fragmented. On the other hand, in the top-down approach, the protein is extracted from cell lysates, separated by liquid chromatography or gel electrophoresis, and then analysed in a mass spectrometer [100,101].

A standard approach in proteome analysis is the use of two-dimensional gel electrophoresis, multidimensional liquid chromatography, tandem mass spectrometry (MS/MS), and database-searching algorithms. The electrophoretic approaches used to analyse the salivary proteome can be distinguished by sodium dodecyl sulphate–polyacrylamide gel electrophoresis (SDS-PAGE) and isoelectric focusing (IEF). An analysis method that uses a combination of these four techniques is often referred to as shotgun proteomics or multidimensional protein identification technology (MudPIT) [84,92]. After gel separation, the proteins are excised and digested with a protease, for example, trypsin, and the masses of the fragments are measured in a mass spectrometer and confirmed in a tandem mass spectrometry analysis (MS/MS). The obtained results are compared with the theoretical predictions available in the databases. The development of LC and MS/MS methods provides an increased ability to the identification of proteins and peptides from complex biological samples [84,102].

#### 2.1.1. General Approaches on Mass Spectrometry Analysis of the Salivary Proteome

**Figure 4:**
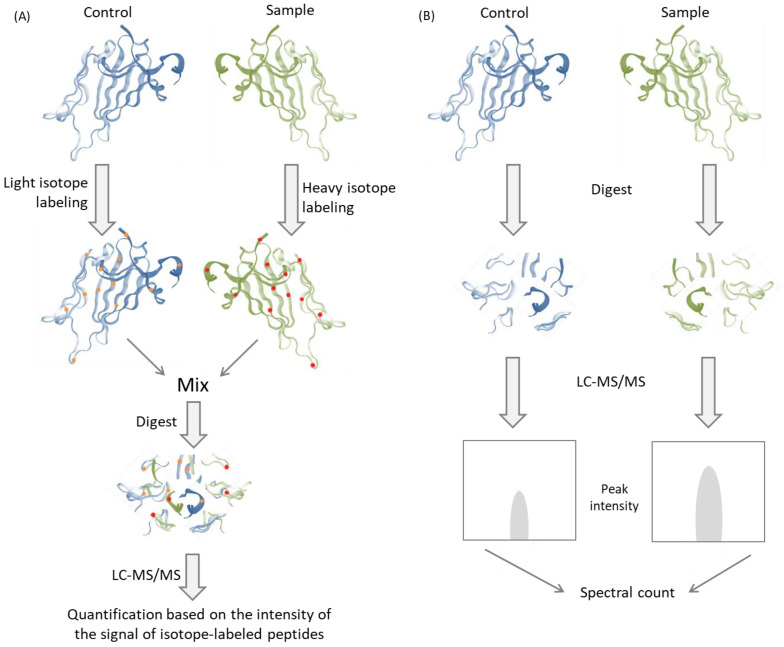
Schematic presentation of two approaches of quantitative proteomics. (**A**) Shotgun isotope labelling method. Samples are labelled with light or heavy isotope, combined and analysed by LC-MS/MS method. Based on the intensity ratio of isotope-labelled pairs of peptides, quantification is performed. (**B**) Label-free quantitative proteomics. Samples are digested and analysed in individual LC-MS/MS experiments. Based on the comparison of peak intensity of the same peptides, quantification is performed [103].

Liquid chromatography coupled with mass spectrometry, in addition to the identification of the peptides and proteins in biological samples, may also be used for the analysis of the concentration of given compounds. In quantitative analysis, two approaches can be distinguished: label-free mass spectrometry and labelling mass spectrometry (Figure 4). For the label-free method, the typical approach is peak intensity-based comparative LC-MS and spectra count-based LC-MS/MS. However, due to possible mistakes caused by variations in the LC and MS yield, these experiments must be carefully controlled. However, the availability of data processing software enables the automatic detection and analysis of peptides from multiple measurements simultaneously, making this technique a highly efficient tool for the detection of molecular biomarkers of disease states [103].

Lately, quantification methods based on mass spectrometry with the use of stable isotope labelling have gained increasing popularity. Tagging allows for the creation of a specific mass tag that can be recognised by the mass spectrometer, which is the basis for quantification. The concentration is calculated from the ratio of the intensities of the pairs of isotopically labelled peptides [104,105]. Although the non-labelled method is more efficient in terms of identified and quantified proteins, the labelling methods have the advantage of a better reproducibility and greater precision [106]. The enzymatic labelling of proteins with ^18^O_2_ is a common strategy in proteomics due to its simplicity and versatility. This method has some disadvantages: labelled oxygen may be backexchanged and the isotopic peaks of labelled and unlabelled peptides can overlap. An alternative to this method is to carry out enzymatic digestion, for example with trypsin (with and without H_2_^18^O), and react the samples with a ^13^C-labelled pyrylium salt [107]. When a peptide is labelled with zero or four ^13^C atoms during MS/MS analysis, it forms a unique reporter ion, allowing for a relative quantification. This method solves the problem of a reverse exchange and reduces the overlapping of isotope peaks.

Recently, we have characterised the methods for designing protein labelling reagents and their application in quantitative proteomics [106]. In general, the structure of isobaric reagents is composed of three groups. The first group is the reporter group, which is readily released from the labelled peptide during collision-induced dissociation (CID). Another component of the isobaric reagent is an isotopically substituted balancing group. Its presence provides the total mass of the reporter ion, and the balancing group is the same in all reagent versions. The last part of the reagent is the reactive group responsible for the selective reaction of the reagent with selected functional groups in the peptide [106]. The peptide derivatisation strategy proposed by us was applied in the proteomics investigation of the canine [108], equine [109], and human [110,111] urine samples, making their reliable and ultrasensitive analysis by mass spectrometry possible.

The most commonly used reactive group is the succinimidyl ester, which is able to undergo a selective reaction with the amino groups in peptides and due to this reaction, a stable amide bond is formed [112]. This group of compounds includes, for example, unlabelled (H_4_) and isotopically labelled (D_4_) nicotinyl-*N*-hydroxysuccinimide, which when applied for the *N*-terminal nicotinylation of peptides for their quantification gives a difference of 4 Da [113]. Isobaric tags for relative and absolute quantification (iTRAQ) have also been described [114]. The protein reactive group in these tags is *N*-hydroxysuccinimide ester. An alternative to iTRAQ is the idea of tandem mass tags (TMTs). An amino group is a reactive group in the tags of the TMTs type that allows for a modification of the primary amines [115]. Another reagent with a reactive group directed to the *N*-terminus and the ε-amino group of lysine is *N,N*-dimethylleucine-based tags [116].

Another group are tags with different reactive moieties, such as pentafluorophenyl esters [117], dimethoxytriazine esters [116], and groups selective for the thiol group, for example, iodoacetamide [118]. One of the described tags containing iodacetamide as a reactive group was the Isotope Coded Affinity Tag (ICAT) [119], which also contains biotin and 8 heavy deuterium atoms. This tag was used for the selective modification of thiopeptides and for quantification in the cysTRAQ method [120]. 

Other reagents for the modification of thiopeptides are iodoTMT zero and iodoTMT sixplex, which are commercially available and are commonly used for the specific detection and quantification of protein *S*-nitrosylation [121]. There are also reports of the use of tags with groups targeting to the carbonyl moiety of proteins, such as substituted hydrazines [122] and hydroxylamines [123].

#### 2.1.2. Improvement of the Mass Spectrometry Analysis

The use of isotopically labelled reagents is an important aspect of quantitative proteomics analysis by mass spectrometry. The second important issue is the sensitivity of detection. Although the LC-MS method is considered to be the most versatile and sensitive for proteomic analysis, there may be some limitations in the analysis of trace amounts of peptides [106]. 

**Figure 5:**
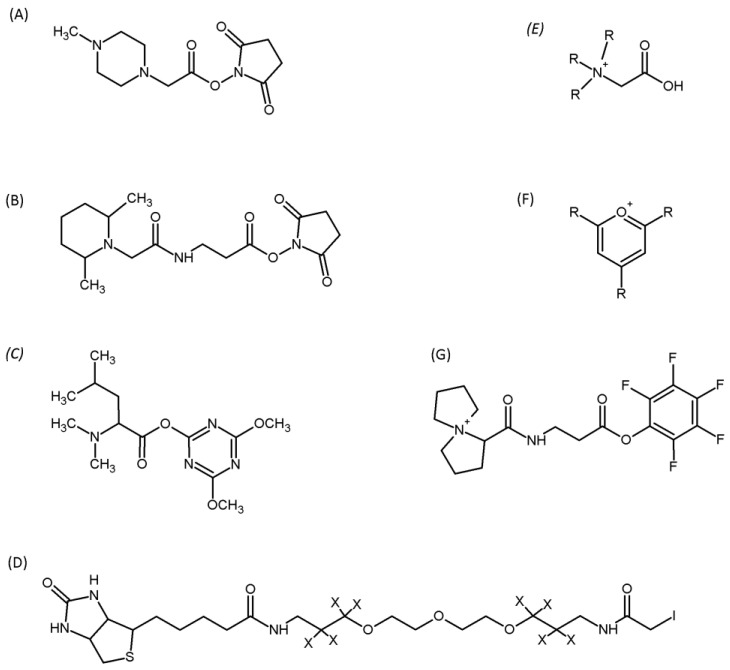
Exemplary structures of compounds used to modify peptides and proteins for their sensitive LC-MS analysis. (**A**) Isobaric tags for relative and absolute quantification (iTRAQ); (**B**) Tandem mass tags (TMTs); (**C**) N,N-dimethylleucine based tag; (**D**) Isotope Coded Affinity Tag (ICAT); (**E**) N,N,N-trialkylglycine; (**F**) 2,4,6-trisubtituted pyrylium salt; (**G**) 5-azoniaspiro[4.4]nonyl (ASN+) scaffold.

This limitation is the low ionisation efficiency (by protonation or deprotonation) and the low ion stability in the MS experiment. To overcome this problem, it is necessary to introduce appropriate groups to increase hydrophobicity [124] and groups with a strong proto-acceptor character [125,126], or those containing a stable charge resulting from the chemical structure such as quaternary sulfonium [127,128], phosphonium [129], or ammonium [130] groups (Figure 5). The presence of a quaternary ammonium group increases the ionisation efficiency of the analysed compound and controls fragmentation, facilitating the MS/MS analysis [131]. 

Recently, we have developed an application of the *N,N,N*-trialkylglycine groups as ionisation enhancers with a quaternary ammonium group in peptide conjugate synthesis for sensitive analysis (attomole level) [132]. Since the development of an efficient method for the hydrogen–deuterium exchange of α-C hydrogens in *N,N,N*-trialkylglycine, the potential of using the developed ionisation tags for peptide analysis has been further increased. [133]. Therefore, their use brings several benefits. These ionisation markers not only lower the limit of detection of the peptides, but they can also be easily converted into their isotopologues for quantitative analysis. 

Other promising ionisation tags with a quaternary ammonium group are compounds based on 2,4,6-trimethylpyryl and 2,4,6-triphenylpyryl salts. The use of a pyrylium salt allows for the selective and efficient derivatisation in solution of the ε-amino groups of lysine, and therefore of peptides containing this amino acid residue. Moreover, the constant positive charge of the pyridinium group increases the ionisation efficiency and enables the identification of trace amounts of the peptide. An abundant protonated ion can be generated by 2,4,6-trisubstituted pyrylium salt, which can be used for multiple reaction monitoring (MRM) analysis [134]. 

The 5-azoniaspiro[4.4]nonyl (ASN+) scaffold is another group of ionisation tags that improve the analysis by mass spectrometry. The tag from this group is, for instance, 1-{[3-oxo-3-(pentafluorophenoxy)propyl]carbamoyl}-5-azoniaspiro[4.4]nonane, which may react selectively with the amino and/or thiol group of the peptides. When collision-induced dissociation experiments are performed, the developed tag is stable, and the CID of the resulting peptide ions generates a dominant series of Y-type fragmentation ions with high sequence coverage. This ionisation tag was successfully used to derivatise the digested model proteins ubiquitin and bovine serum albumin, and the isotopically labelled derivatisation reagent analogue was used for a comparative quantification by LC-ESI-MS [135].

In addition to the modification of the amino group of the *N*-terminus of peptides or the ε-amino group of the lysine side chain, an important and attractive modification target for proteomic analysis is the thiol group of the cysteine side chain. The thiol group is the most nucleophilic functional group found in peptides, which significantly increases the selectivity of the modification. Furthermore, cysteine is one of the least abundant amino acids in the protein sequence, and therefore the targeted modification significantly reduces the complexity of the proteome [136,137]. The above features are the basis for numerous studies aimed at enriching and modifying thiopeptides. The developed methods of the enrichment of cysteine-containing peptides can be divided into an enrichment in solution or on a solid support and among them, the methods of enrichment through a thiol–disulphide exchange [138], thia-Michael addition [139], and charge derivatisation [140].

Salivary proteome mass spectrometry analysis aimed at identifying cysteine-containing proteins has been described for both human salivary and insect proteome. A previously unknown protein (ACYPI39568) rich in cysteine (14 residues) belonging to the family of aphid-specific proteins was identified in the saliva of aphids secreted into the phloem of plants during an infestation. An identification was performed using the matrix-assisted laser desorption ionisation coupled to time-of-flight mass spectrometry (MALDI-TOF) and circular dichroism (CD) methods [141]. Cysteine-containing phosphoproteins were identified in submandibular-sublingual saliva and distinguished from other phosphoproteins by the presence of half-cystine [142]. Cysteine-containing phosphoproteins have also been identified in parotid saliva and tears and quantified using rocket immunoelectrophoresis [143].

### 2.2. Salivary Proteome Studies

#### 2.2.1. Characterisation of Salivary Proteome

In 2005, Hu et al. [98] conducted a global analysis of the salivary proteome to identify proteins and peptides that help maintain oral homeostasis. The study employed shotgun proteomics through prefractionating proteins: small proteins were separated by liquid chromatography and large proteins were first digested with trypsin and then separated by liquid chromatography. Afterward, two-dimensional gel electrophoresis and mass spectrometry analyses were performed. The obtained results were compared with the data available in databases (MASCOT and Pro ID program) to identify the proteins. Using the presented proteomics approach, 309 human salivary proteins have been described. These findings indicate that the presented approach can be used to monitor the protein composition of saliva in various disease states.

In 2009, Ambatipudi et al. [144] conducted comprehensive proteomics profiling of the saliva of women from two age groups (20–30 and 55–65 years) due to the higher incidence of autoimmune diseases affecting the function of the salivary gland [145]. To reduce the sample complexity, saliva was fractionated by hydrophobic charge interaction chromatography (HCIC). This was followed by mass spectrometry analysis and the quantification of protein expression by MS using multidimensional protein identification technology (MudPIT). The International Protein Index (IPI) protein database was searched with the use of the ProLuCID algorithm and the results were assembled and filtered using the DTASelect. For protein annotation, UniProtKB/Swiss-Prot databases were used. The selected results were further confirmed by Western blot. The study identified 532 proteins, including 266 commons to both age groups. Most of the identified proteins are involved in the immune response, and proteins such as lysozyme and histatin-1 were more abundant in women between the age of 55 and 65. Therefore, the expression of salivary protein was found to be age-dependent, which is important in the search for potential molecular biomarkers of diseases. Research into the salivary proteins associated with diseases specific to women provides the basis for the identification of salivary proteins [144].

In 2010, Salih et al. [146] characterised phosphoproteins in oral fluids, including the abundant protein components of saliva. The study involved five volunteers without the impairment of the salivary gland function. Trypsin digestion was performed on the collected saliva samples. Dithiothreitol was used to derivatise the tryptic peptides containing phospho-serine or phospho-threonine. Before the derivatisation, the cysteine residues were blocked by iodoacetamide and enzymatic *O*-deglycosylation. The modified peptides were enriched by covalent disulphide–thiol exchange chromatography followed by nanoflow liquid chromatography coupled with tandem mass spectrometry (LC-ESI-MS/MS) analysis. The obtained MS/MS spectra were searched against several databases, including Uniprot, Swiss-Prot, TreMBL, and PIR, using Bioworks 3.3.1 software and the SEQUEST search engine. In the research, 65 phosphoproteins were identified, about 80% of which have not been previously described. The conducted analysis of the salivary phosphoproteome is the basis for extending research on the identification of disease states by the composition of the salivary phosphoproteome.

**Figure 6:**
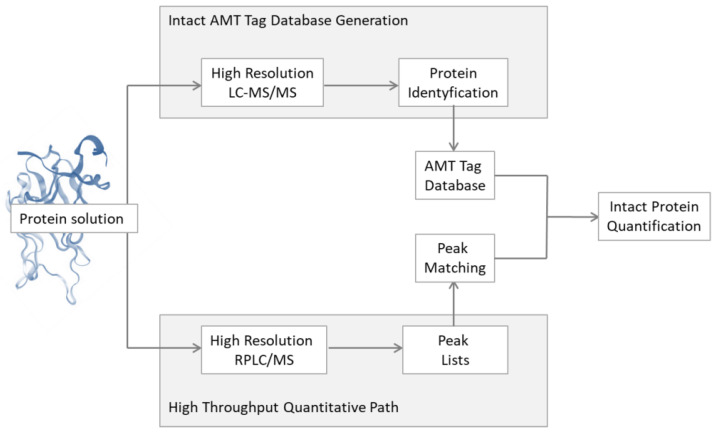
Schematic presentation of the quantification withe use of the accurate mass and time AMT Tag approach.

In 2014, Wu et al. [147] conducted a top-down LC-MS/MS analysis of the salivary proteome, which characterised 20 human salivary proteins and 83 proteoforms containing a wide range of polymorphic isoforms, including proteins such as the *O*-glycosylated acidic protein, *N*-glycosylated protein, prolactin inducible protein, and β-2 microglobulin. The identified proteins provided a comparative database of intact salivary proteoforms for the quantification of salivary proteins from six healthy volunteers. The samples of human parotid and sublingual salivary gland secretions were compared using reverse-phased liquid chromatography (RP-LC) coupled with the FT-ICR mass spectrometer, and the profiles of the secreted proteoforms were separated with a high reproducibility. For the quantification and analysis of differentially abundant proteoforms, an intact AMT tag approach was developed (Figure 6). The LC-MS analysis approach with an intact protein showed the high application potential for discriminating between healthy and diseased states through the rapid and accurate identification of biomarkers. The protein identification was achieved by searching every MS/MS spectrum against the protein sequence databases-annotated top-down human database and UniProt FASTA.

The oral cavity is the primary entry point for pathogens and microbial organisms. Saliva is responsible for maintaining homeostasis in the oral cavity. Therefore, disturbances in the salivary secretion process and changes in the oral microbiome may cause tooth decay or respiratory infections. [148]. In 2016, Grassl et al. [149] mapped the deep salivary proteome of eight healthy volunteers at different time points. The analysis was performed using reverse-phase liquid chromatography to fractionate the samples, followed by LC-MS determination. To prevent the involvement of peptides common to humans and bacteria, a “split by taxonomy id” method was developed, which enabled the precise identification of microbial proteins. A total of 5500 human proteins and 2000 microbial proteins from 50 different types of bacteria were quantified in the microgram samples of the protein after fractionation. Additionally, during eating and the brushing of teeth, drastic changes in the oral microbiome were observed. The obtained results were compared with next-generation sequencing data (Human Microbiome Project) and with MALDI-TOF mass spectrometry, and a high agreement was obtained. The study indicated that both shotgun proteomics and advanced technology could be used simultaneously to characterise and quantify the interaction of the human immune system with bacteria and for the clinical diagnosis of saliva. 

#### 2.2.2. Studies of the Salivary Proteome for Cancer Detection

**Figure 7:**
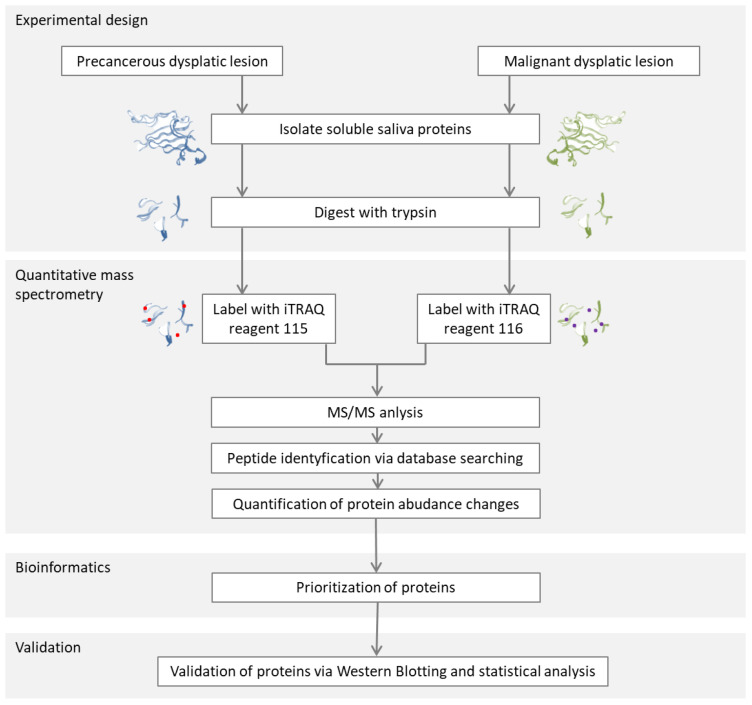
Schematic representation of the strategy of salivary proteome analysis from people with precancerous dysplastic lesion and malignant dysplastic lesions [150].

Because of the non-invasive nature of collecting biological material, tests for the presence of proteins whose abundance in saliva suggests the occurrence of precancerous changes or malignant changes are currently being sought. (Figure 7). To discover these proteins, in 2010, de Jong and co-workers [150] performed advanced quantitative proteomics based on the mass spectrometry analysis of saliva samples from eight people with precancerous and malignant lesions. The MS/MS spectra were searched using SEQUEST software against a non-redundant human protein sequence database. The matched peptides were filtered by expected isoelectric points. Using bioinformatics methods (Ingenuity Pathway Analysis (IPA)) software, the proteins were identified as potential biomarkers and validated by Western blotting. This analysis revealed an increased abundance of actin and myosin in people with malignant lesions. On a saliva sample from twelve other people with precancerous lesions and another group of twelve people with malignant lesions, the analysis was further confirmed. The values of sensitivity/specificity for distinguishing the types of lesions were 100%/75% for actin and 67%/83% for myosin. The abundance of myosin and actin in saliva makes it possible to distinguish lesions with a high specificity and sensitivity. Further studies of the described method may lead to the development of non-invasive saliva tests for the early detection of oral cancer. 

In 2016, Xiao et al. [151] conducted a study to identify gastric cancer biomarkers in saliva. The assay involved patients diagnosed with gastric cancer and a matched control group, and the assay was performed using tandem mass tracers (TMT). The labelled peptides were fractionated by cation exchange chromatography followed by LC-MS/MS analysis. The obtained spectra were used for a protein identification and SEQUEST interfaced with Proteome Discoverer was used for the protein database search against the IPI human database. The proposed procedure allowed for the identification of 500 proteins. A significant difference in the expression of eight proteins was observed between gastric cancer patients and controls. Three of the five selected proteins (triosephosphate isomerase, cystatin B, and a protein deleted in malignant brain tumours 1) were then successfully verified by an ELISA. These proteins were found to be biomarkers for gastric cancer, and their combined presence could lead to a sensitivity of 85% and 80% specificity. The results of the described study are promising and, after being validated on a large scale, may be an alternative method for the detection of gastric cancer in screening tests.

In 2016, Delmonico et al. [152] conducted a study of the proteomics profile of the saliva and plasma of women diagnosed with fibroadenoma (benign lesions) or infiltrative ductal carcinoma malignant. One-dimensional gel electrophoresis was performed, and the gel fragments were digested with trypsin. Afterward, liquid chromatography coupled with mass spectrometry (nLC-Q-TOF technology) was used for the analysis. The patient’s saliva and plasma were analysed by a combination of inter/intra-group analyses, and significant quantitative and qualitative differences were revealed. The obtained MS/MS results were analysed using Mascot. The tandem mass spectra in the MSDB proteins and the NCBInr database were correlated with MASCOT online software, which was used to identify proteins. Changes in immune, signalling, and molecular transport pathways turned out to be the most representative of the proteomic profile of plasma and saliva. The main proteins with a differentiated expression in the saliva of the research group in comparison to the control group were α-2-macroglobulin and ceruloplasmin, in the case of fibroadenoma, as well as infiltrative ductal carcinoma. The plasma levels of α-2-macroglobulin and ceruloplasmin were increased, while the levels of proteins such as the vitamin D binding protein, hemopexin, and haptoglobin were decreased compared to the levels of these proteins in the control group. The same expression variability was observed in other studies [153,154] using isotopically labelled proteins in a group of cancer patients in comparison to the healthy control group. The saliva samples were digested with trypsin, labelled with iTRAQ reagent, and analysed by reversed-phase capillary chromatography on a mass spectrometer equipped with LC-Packings HPLC. These studies suggest that saliva is rich in by-products of an oncogenic expression.

In 2021, Ishikawa et al. [155] performed a study to identify biomarkers that differentiate oral cancer patients from healthy individuals. The study involved 39 patients diagnosed with oral cancer, and the control sample consisted of 31 healthy volunteers. The LC-MS/MS method was used for the analysis, followed by statistical analysis to distinguish between oral cancer patients and healthy individuals. A multiple logistic regression (MLR) model was developed to assess the discriminatory ability of multiple marker combinations. For data analysis, Proteome Discoverer software was used to search for the MS/MS spectra against the UniProt human database and the raw data were processed using the Mascot database search engines. Twenty-three different proteins were identified for cancer patients and healthy individuals. Six of these proteins were selected (α-2-macroglobulin-like protein 1, hemoglobin β subunit, corulin, kininogen-1, transmembrane protease serine 11D, Ig ĸ chain V-II region Vĸ167) and used for the MLR model. The results indicated that these proteins could be considered as potential biomarkers for oral cancer screening.

Periodontal disease, which affects the tissues supporting the teeth, is a disease where the proteins found in saliva may serve as biomarkers. Identifying these proteins could enable the early diagnosis and easy monitoring of the disease. In 2018, Bostanci et al. [156] performed a study of the salivary proteome in people with periodontitis and gingivitis and healthy volunteers. A quantitative proteomics approach using open-ended label-free quantitative analysis was used (protein digestion with trypsin, nanoLC-1D chromatographic separation, LC-MS analysis, and statistical analysis). The raw LC-MS data were normalised and aligned against an in-house-built database, created using human, bacterial, and fungal species, with the use of the Mascot search engine. For validation, a selected-reaction monitoring (SRM) assay was performed to identify and determine the relative abundance of the proteins. In the study, 119 proteins were identified, which may indicate the difference between a person with periodontitis or gingivitis and a healthy person. Sixty-five of these proteins were selected for SRM analysis and 60 of them were successfully quantified. The statistical analysis led to the selection of five proteins, which the presence of may indicate periodontal disease with a great probability. These proteins may be new potential biomarkers for periodontal diseases due to their specificity and sensitivity.

#### 2.2.3. Studies of Salivary Proteome in Type 1 and Type 2 Diabetes

**Figure 8:**
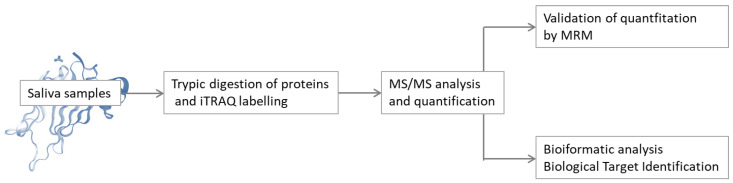
Schematic representation of the proteomic and bioinformatics analysis of saliva [157].

In 2018, Pappa et al. [157] conducted a study of the salivary proteome of children and adolescents with type 1 diabetes and healthy individuals. The collected protein samples were digested by trypsin, and then the tryptic peptides were isotopically labelled using iTRAQ. High-pH RP C18 fractionation and LC-MS and LC-MS/MS analysis were then performed for the identification and quantification of the resulting peptides. The collected MS/MS spectra were searched against a UniProt Fasta database. The study also included MRM quantification validation and bioinformatics analysis (Figure 8). It was found that the proteomic profiles of the saliva of people with proper glycaemic control and healthy people are comparable. In contrast, in the saliva of people with poor glycaemic control, the proteins involved in processes related to diabetic pathologies, such as endothelial inflammation and damage, have been identified, and the expression of these proteins is different compared to healthy patients or patients with normal glucose levels. In addition, a preventive therapeutic approach was proposed based on bioinformatics analysis.

This was not the first research that targeted the proteome of the saliva of people with diabetes. In 2009, Rao et al. [158] identified 65 proteins with different expressions in the saliva of people with type 2 diabetes compared to pre-diabetes patients and healthy volunteers. The characterisation of the salivary proteome was performed using the 2D-LC-MS/MS method, and a quantification was performed without isotope labelling. Tandem mass spectra were searched against composite protein databases (Swiss-Prot and TrEmbl). In 2010, Cabras et al. [159] identified peptidomimetic modifications in the saliva of people with type 1 diabetes. The collected saliva samples were analysed by reversed-phase HPLC-ESI-MS. In 2013, Bencharit et al. [160] characterised the proteomic changes associated with hyperglycaemia by label-free quantitative proteomics.

#### 2.2.4. Studies of Salivary Proteome during Pregnancy

In 2020, Dey et al. [86] performed a study of changes in the longitudinal proteome of saliva during 6 to 29 weeks of pregnancy to observe protein expression. The study included women with undisturbed courses of pregnancy. A saliva-specific library was created by combining one-dimensional SDS-PAGE, LC-MS/MS, and RP-C18-HPLC followed by a label-free quantification in the Sequential Window Acquisition of All Theoretical Mass Spectra (SWATH-MS). The collected spectra were analysed and searched against the UniProt human reference protein database. The study quantified 65 proteins, the concentration of which changes as pregnancy develops. It has been determined that these proteins are involved mainly in the metabolism, host defence mechanism, and maternal immune modulation. Subsequently, the LC-MS-MRM analysis of the twelve selected proteins was performed, which confirmed the expression pattern determined by the SWATH-MS analysis. The presented results constitute the first step in the comparative analysis of the salivary proteome in the undisturbed course of pregnancy and the adverse pregnancy outcomes. Therefore, it may contribute to the direction of specific molecular pathways in the discovery of biomarkers.

#### 2.2.5. Studies of Salivary Proteome of Patients with Sjögren’s Syndrome

In 2021, Das et al. [161] analysed the saliva and tears proteome of people with Sjögren’s syndrome (dysfunctional mucous membranes) and compared it to the proteome of a control sample. The collected samples were hydrolysed with 1% SDS and were subjected to trypsin digestion; then, the samples were isotopically labelled on the α- and ε-amino groups of peptides and were separated by chromatography using C18 columns. Afterwards, the samples were subjected to LC-MS analysis and the obtained results were compared with proteomics databases (human UniProt protein database and PRIDE database). In order to study the protein–protein interaction, a bioinformatics analysis was performed (the Search Tool for the Retrieval of Interacting Genes (STRING) database was used). In total, 153 proteins, including 45 downregulated proteins, and 18 proteases and protease inhibitors were identified in the saliva, which were expressed differently in patients with Sjögren’s syndrome and in healthy people. Modified protease activity can initiate inflammation and cause a pro-inflammatory response in Sjögren’s syndrome. Additionally, during the study, quantification with the immunoassay of proteoglycan 4, involved in maintaining homeostasis and regulating inflammatory signalling, was performed. The observed changes in the protein expression in people with Sjögren’s syndrome may be the first step in the identification of new drug targets; a particularly promising target is proteoglycan 4, which was first identified in saliva. The summary of the salivary proteome studies is presented in Table 2.

#### 2.2.6. Studies of Salivary Microbiome

In 2008, the term “salivaomics” was introduced to emphasise the amount of research and development of knowledge about the components of the saliva, the proteome, metabolome, as well as transcriptome or microbiome, which gives new opportunities for the diagnosis and monitoring of several diseases [162,163].

Recently, many in vitro proteomics studies have been conducted on periodontal diseases and periodontal pathogens. These studies were summarised in a review prepared by Nguyen and co-workers [164]. The most commonly studied species of pathogens are *Porphyromonas gingivalis* and *Aggregatibacter actinomycetemcomitans*. *Porphyromonas gingivalis* releases virulence factors and this microorganism is a major pathogen of periodontitis. Proteomics studies have shown that *A. actinomycetemcomitans* and *P. gingivalis* produce multiple outer membrane vesicles that contain numerous effector proteins. These proteins are internalised into human host cells. In vitro proteomics studies enable the analysis of the host’s response to microbial proteins. For example, several studies have tested bacteriocins (protein or peptide compounds) for their ability to inhibit bacterial colonisation. The advantage of in vitro proteomic studies is also the ability to assess the impact of a more complex environment and to study the many species of microorganisms simultaneously.

In chapter 2.2.1, it was mentioned that Grassl et al. [149] mapped the deep salivary proteome using the LC-MS method. However, due to the significant role of the oral microbiome, the detection of bacteria in the deep saliva proteome was also investigated. For this purpose, complete Uniprot protein sequences of bacterial species identified by 16S rRNA sequencing were taken. The result was a database, which was approximately 11 times larger than the human HMP database. The identified bacterial proteomes were compared with whole-genome sequencing data (HMP) in principal component analysis (PCA) and a heatmap. Despite the origin of the samples from different individuals, the obtained proteomics data were closely co-located with the oral microbiome, consistent with previous findings showing that the oral microbiome has a relatively low diversity.

Recently, Pappa et al. [165] analysed the human and microbial salivary proteome of children with molar incisor hypomineralisation (MIH) to identify salivary biomarkers of pathology. The study involved 10 children diagnosed with MIH and 10 healthy children (control sample). After the sample’s preparation, a mixture of tryptic peptides was separated by nano-liquid chromatography and analysed by mass spectrometry and tandem mass spectrometry. In order to analyse the host proteins, the obtained results were compared with the data available in the FASTA database using Proteome Discoverer 2.4. A statistical evaluation was also performed using the PCA test and clustering (heat map) functions. For metaproteomic analysis, the database was searched using MetaLab software. The identified peptide sequences were subjected to taxonomic analysis with the use of the lowest common ancestor (LCA) algorithm. The conducted research allowed for the identification of 1515 proteins with an indication of the diversity between the two research groups. Furthermore, the performed statistical analysis enabled the identification of 142 proteins with different expressions. The study showed a distinct difference between children with MIH and healthy children, which demonstrates the disorder of regulation of the inflammatory mechanisms and the mechanisms of the defence response to bacteria in people with molar incisor hypomineralisation. The bacterial proteome analysis showed a lower microbial species diversity, underscored by a microbial imbalance in MIH pathology.

## 3. Conclusions

The investigation of drugs, their metabolites, and the peptide or protein molecular biomarkers of biological fluids is usually limited due to their complex nature. Saliva is one of the excellent biofluids which is characterised by a high availability, easy sampling, and preparation for further analysis. Among the salivary metabolomics and proteomics strategies, mass spectrometry coupled with separation and fragmentation techniques has become the method of choice in qualitative and quantitative analysis. The solutions implemented so far enabled the development of methods for identifying drugs, their metabolites, peptides, and proteins which may serve as molecular biomarkers. However, many tasks remain to be solved, including the sensitivity of detection and the unambiguous identification of compounds by fragmentation or quantitative analysis. These problems can be avoided or minimised by using the charge derivatisation strategy of the samples or obtaining isotope-labelled standards by a simple and cost-efficient isotope exchange reaction in biomarker analysis, which are the future prospects. Most of these strategies have been applied in proteomics studies and now their applicability is also being tested and implemented in salivary metabolomics. Those two omics are related by many aspects, including protein degradation and expression, their post-translational modification, and cell signaling. A combination of proteomics and metabolomics data to qualify and quantify changes in metabolites and proteins, by the identification of novel biomarkers, may advance our knowledge of pathophysiological states and their mechanisms. Therefore, the improvement of the sensitivity, accuracy, precision of measurements, and their reproducibility is a significant task in those two omics techniques which may create an excellent tool for the identification of new molecular biomarkers for a clinical diagnosis and/or forensic investigation. The presented review provides further knowledge of salivary metabolomics and proteomics for a broader audience.

## Figures and Tables

**Table 1:** Methods of different metabolites detection in saliva.

Metabolite	Analytical Technique	Quantitative Analysis	Literature
Cortisol	LC-MS/MS	Internal standard cortisol-d_4_	[51,52,53,54,55,56,57,58,59,60]
*p*-cresol sulphate and indoxyl sulphate	LC-MS/MS	*p*-cresol sulphate-d_7_ and indoxyl sulphate-d_4_	[61]
Steroids	LC-MS/MS (with multiple reaction monitoring)	Internal standard	[63]
Creatinine	LC-MS/MS	Isotope-labelled internal standard creatinine-d_3_ and creatinine-d_5_	[67,68,69,70]
Lacosamide	HPLC	internal standard	[71]
Levetiracetam	HPLC-MS/MS	Internal standard fluconazole	[72,73,74,75]
Armodafinil	LC-MS/MS	Internal standard armodafinil-d_3_	[76,77]
Cyclosporine A	LC-MS/MS	Internal standard cyclosporine A-d_3_	[79]
Carbamazepine and its metabolite	UHPLC	Internal standard chlordiazepoxide	[80]
sTRAIL	LC-MS/MS	Internal standard ^15^N-labelled hrTRAIL	[81]

**Table 2:** Summary of the salivary proteome studies.

Type of Study	Analytical Technique	Proteolysis	Database	References
Global analysis	Shotgun proteomics, 2D-GE-MS, LC-MS/MS	Trypsin Digestion	MASCOT and Pro ID program	Hu et al. [92]
Proteomic profiling of the saliva of women	HCIC MS (MudPIT), Western blot	Trypsin Digestion	EBI International Protein Index	Ambatipudi et al. [144]
Phosphoproteins identification	LC-ESI-MS/MS	Trypsin Digestion	Uniprot, Swiss-Prot, TreMBL, PIR	Salih et al. [146]
Proteins identification	Top-down LC-MS/MS	-	official_human_TD, UniProt FASTA.	Wu et al. [147]
Human and bacteria proteins identification	LC-MS, shotgun	Lysis buffer (1 % sodium dodecyl carbonate (v/v), 10 mM tris (2-carboxyethyl) phosphine, 40 mM 2-chloroacetamide, 100 mM Tris buffer pH 8.5), Trypsin Digestion	UniProt, HMP	Grassl et al. [149]
Malignant changes	MS, bioinformatics, Western blotting	Trypsin Digestion	Non-redundant human protein sequence database	de Jong et al. [150]
Gastric cancer biomarkers identification	TMT, LC-MS/MS, ELISA	Trypsin Digestion	IPI human database.	Xiao et al. [151]
Fibroadenoma or infiltrative ductal carcinoma malignant	LC-MS	Trypsin Digestion	MSDB database	Delmonico et al. [152]
Oral cancer biomarkers identification	LC-MS/MS,	Lysis buffer	UniProt, Mascot	Ishikawa et al. [155]
Proteome characterisation in periodontitis and gingivitis	nanoLC-1D, shotgun LC-MS, statistical analysis, SRM	Trypsin Digestion	In-house-built database	Bostanci et al. [156]
Type 1 diabetes	labelling with iTRAQ, LC-MS, LC-MS/MS, MRM, bioinformatics	Trypsin Digestion	UniProt Fasta	Pappa et al. [157]
Type 2 diabetes	2D-LC-MS/MS	-	Swiss-Prot, TrEmbl	Rao et al. [158]
Characterisation of salivary proteome during pregnancy	LC-MS/MS, SWATH-MS, LC-MRM	Trypsin Digestion	UniProt	Dey et al. [86]
Sjögren’s syndrome	LC-MS, bioinformatics	Trypsin Digestion	UniProt, PRIDE, STRING	Das et al. [162]

## Data Availability

The data presented in this study are available in the article.
